# Enterobacteriaceae and *Salmonella* contamination of wild boar (*Sus scrofa*) carcasses: comparison between different sampling strategies

**DOI:** 10.1007/s10344-021-01531-0

**Published:** 2021-09-25

**Authors:** Silvia Bonardi, Cesare Tansini, Antonio Cacchioli, Laura Soliani, Luca Poli, Luca Lamperti, Margherita Corradi, Stefano Gilioli

**Affiliations:** 1 Department of Veterinary Science, University of Parma, Strada del Taglio 10, 43126 Parma, Italy; 2 Risk Analysis and Genomic Epidemiology Unit, Istituto Zooprofilattico Sperimentale Della Lombardia E Dell’Emilia-Romagna, Sezione di Parma, Strada dei Mercati 13/A, 43126 Parma, Italy; 3 Management Body for Parks and Biodiversity “Emilia Occidentale”, 43038 Sala Baganza (PR), Italy

**Keywords:** Wild boar, Game-handling establishment, Enterobacteriaceae, *Salmonella*, *Yersinia*

## Abstract

During 2020, a total of 64 wild boar carcasses were tested for Enterobacteriaceae count (EBC), *Salmonella* and *Yersinia enterocolitica* in the abdominal region (i) within 5 h after hunting in the game collection point and (ii) before dressing and processing in the game-handling establishment (GHE) (49 carcasses—average time interval between (i) and (ii): 4.3 days). Because of COVID-19 restrictions, 15 carcasses were transported to a near slaughterhouse (average time interval between (i) and (ii): 2.3 days). Mesenteric lymph nodes (MLNs) were collected and tested for *Salmonella* and *Y. enterocolitica*. Results are shown in relation to sampling A (49 carcasses—GHE) and sampling B (15 carcasses—slaughterhouse). Sampling A: EBC median values were (i) 2.51 log_10_ CFU/cm^2^ and (ii) 2.79 log_10_ CFU/cm^2^. EBC increase between (i) and (ii) was statistically significant (*p* = 0.001). *Salmonella* prevalence on carcasses varied from (i) 2.0 to (ii) 6.1%. Sampling B: EBC median values were (i) 3.1 log_10_ CFU/cm^2^ and (ii) 3.32 log_10_ CFU/cm^2^. EBC increase between (i) and (ii) was not statistically significant (*p* = 0.191). *Salmonella* prevalence on carcasses varied from (i) 6.7 to (ii) 0.0%. The prevalence (sampling A + B) of lymphatic *Salmonella* carriers was 7.8% (5/64). From carcasses and/or MNLs, the serovars Enteritidis, Typhimurium, Agama, Zaiman and Diarizonae O:50 (z) were detected. *Y. enterocolitica* was never isolated. Long chilling periods prior to wild game processing should be avoided, and carcasses should be tested at GHE rather than after shooting to proper reflect the microbial load of wild boar meat entering the food chain.

## Introduction

Wild boars (*Sus scrofa*) are among the most common wild ungulates in Italy. Their presence has been documented from the north to the south of the country, with higher prevalence through the Apennines mountains (central Italy) (Carnevali et al. 2009). Recently, their density has been estimated to range between 1.37–2.31 animals/km^2^ in the region of the study (Emilia-Romagna region), which is considered a significant density for this species (Pittiglio et al. 2018). Their high reproductive rate (Gethöffer et al. 2007) and omnivorous nature (Chiari et al. 2013), together with gradual desertion of rural areas (Orsoni et al. 2020), strongly contribute to their continuous increase. Therefore, wild boar meat consumption has been implemented along with animal population increasing during the last 30 years (Ramanzin et al. 2010).

Wild boar meat could satisfy several consumer requirements, such as sustainability of local food products and local economies (Orsoni et al. 2020), together with higher meat quality comparing with pig meat, resulting in a lower fat content and a better ratio in polyunsaturated fatty acids and saturated fatty acids (Barbani et al. 2011; Sales and Kotrba 2013). In addition, hunted-game animals live their entire life in their natural environment, fulfilling all species physiological and ethological needs as opposed to farmed animals, whose intensive farming practices could be perceived as morally questionable by consumers (Bruckner 2007). Apart from ethical considerations, hunted-game animals do not receive any kind of veterinary treatment during their life, resulting in the most “antibiotic-free” meat humans may ever eat.

Wild boars may carry zoonotic pathogens, such as *Salmonella* spp. and *Yersinia enterocolitica* (Bonardi et al. 2019; Fredriksson-Ahomaa et al. 2020; Sannö et al. 2018; Wacheck et al. 2010). *Salmonella* spp. was assessed as of high priority in wild boar meat safety assurance (EFSA 2013) and it is considered a relevant biological hazard in wild animals (Gortázar et al. 2007). Nevertheless, the role of wild boar meat in the epidemiology of human salmonellosis and yersiniosis has not been studied so far. In 2019, salmonellosis and yersiniosis were the second and fourth most frequently reported zoonoses in the EU, with notification rates of 20.0 cases/100,000 population and 1.7 cases/100,000 population, respectively (EFSA and ECDC 2021).

To protect human health, safety, traceability, labelling and official controls of wild game meat are ensured by the European Union (EU) Regulations No. 178/2002, 852/2004, 853/2004, 1169/2011, 2017/625 and 2019/627, which are the same legislative acts in force for farmed food-producing animals. Since crucial differences exist between animal hunting and farming, specific rules are addressed to wild animals as opposed to farmed species. Therefore, Regulation No. 853/2004 laying down specific rules for food of animal origin states that large wild game carcasses should be examined by a “trained person” for absence of macroscopic lesions soon after killing. This first examination of hunted large game is often performed in game collection points (GCPs) located in the hunting areas, where carcasses must be eviscerated as soon as possible and transported to a game-handling establishment (GHE) after the removal of stomachs and intestines. At the GHE, postmortem inspection of carcasses and offal is performed by official veterinarians following Regulation No. 2019/627. After processing, large wild game meat should be chilled at maximum 7 °C.

What is still missing for game meat, as opposed to meat of farmed ungulates, are the process hygiene criteria set down by Regulation No. 2073/2005. The absence of legal microbiological criteria for game meat obstacles any kind of hygiene and safety controls by the Competent Authority (CA) and does not support self-monitoring actions by Food Business Operators (FBOs).

The study was planned to test wild boar carcasses after evisceration in the GCP located in the “Boschi di Carrega” Regional Park, Parma province (Northern Italy), and to re-test them before processing at the GHE located 150 km away in Bologna province (Northern Italy). Unfortunately, due to COVID-19 pandemic and travel restrictions, some carcasses were tested in Parma province only, i.e. after evisceration in the GCP and before processing in a swine slaughterhouse located 50 km away. The aims of the study included (i) the analyses of carcasses in the most likely contaminated location (abdominal area) for Enterobacteriaceae count, *Salmonella* spp. and *Y. enterocolitica* to address their hygienic status both soon after killing and before processing in the GHE; (ii) the detection of *Salmonella* spp. and *Y. enterocolitica* in mesenteric lymph nodes to identify carriers’ prevalence; and (iii) the influence of anatomical shooting location on carcasses contamination. Due to the abovementioned modifications in the sampling plan, the study was completed with (iv) the comparison between the hygienic parameters of carcasses processed at a remote GHE vs. carcasses processed at a pig slaughterhouse located in the same hunting province.

## Materials and methods

### Sampling

During 2020, 64 wild boars were analysed for Enterobacteriaceae count (EBC), *Salmonella* spp. and *Y. enterocolitica*. The animals were hunted in Parma province (Northern Italy) during wildlife control plans from January to March and from October to December 2020. At the end of each hunt, the carcasses were transported to a collection point within 5 h of animals’ death. Wild game carcasses are considered expertly shot when the wound is fatal and exclude the abdominal cavity. On the contrary, non-expertly shot animals are shot in the abdomen with severe damage and faecal soiling (Atanassova et al. 2008). In our study, data from shooting location were recorded, dividing the carcasses in two groups: (I) correct shot placement (head, neck, heart, thorax); (II) incorrect shot placement (abdomen).

Sampling A1—game collection point (GCP). In a GCP, authorised by the CA and located in proximity of the hunting territory, 49 wild boar carcasses were tested by swabbing two areas of 100 cm^2^ each in the abdominal wall (right side) after evisceration, but before being transported to the GHE. Inside the GCP, all carcasses were eviscerated in a hanging position by the back legs and not in a lying position on the floor, as could occur when carcasses are eviscerated directly on the field. For all carcasses, the time interval between killing and onset of refrigeration did not exceed 5 h. To remove blood and organic detritus caused by shooting, the inner part of the carcasses was thoroughly washed with running cold potable water before being stored at refrigeration temperature (2 ± 1 °C). Sampling was performed after the washing step to reproduce the microbiological conditions of the carcasses before chilling. The sterile sponges were moistened with 10 ml of buffered peptone water (BPW, Oxoid, Basingstoke, UK) before use. One area was swabbed to be tested for EBC, the other one for *Salmonella* and *Yersinia*. Mesenteric lymph nodes (MLNs) were collected using sterile instruments and placed in sterile containers. Carcass swabs and MLNs were analysed on the day of sampling. Data from anatomical shooting location were recorded and the carcasses were assigned to group I or II.

Sampling A2—game-handling establishment (GHE). After transportation to the GHE, the chilled skin-on carcasses were re-tested by swabbing two areas of 100 cm^2^ of the abdominal wall (left side). Time between evisceration of carcasses and in the GCP and their processing at the GHE varied between 2 and 9 days (average 4.3 days).

Sampling B1—game collection point (GCP). Fifteen wild boar carcasses were swabbed after evisceration and cold water washing as described in sampling A1. MLNs were collected as described in sampling A1. The carcasses were divided into groups I and II based on the anatomical shooting location.

Sampling B2—slaughterhouse (SH). Due to travelling limitations during the COVID-19 pandemic, the 15 wild boar carcasses were transported to a slaughterhouse located 50 km away from the GCP. Carcass swabbing was performed as described in sampling A2. Time between evisceration of carcass in the GCP and processing at the slaughterhouse varied between 1 to 5 days (average 2.3 days).

### Analytical methods

The Enterobacteriaceae count (EBC) was performed following the ISO 21528–2:^2017^ method. Each sponge was diluted in 90 ml of BPW, followed by tenfold dilutions up to 10^−4^. The second sponge was cut into two identical pieces, each one used for the detection of *Salmonella* and *Y. enterocolitica* following the ISO 6759–1:^2017^ and ISO 10273:^2017^ methods, respectively. MLNs were rinsed with sterile water and externally decontaminated with ethanol before being cut in small pieces with sterile instruments. Aliquots of 5 g each were tested for *Salmonella* and *Y. enterocolitica*. Biochemical identification of the isolates was performed by using the API 20E © microsubstrate system (bioMérieux, Marcy l’Etoile, France). *Salmonella* serotyping was performed following ISO/TR 6579–3:^2014^.

Antimicrobial resistance (AMR) of *Salmonella* isolates was tested by the minimum inhibitory concentration (MIC) test using the YEUVSEC3 Sensitre™ plates (ThermoFisher Scientific, Waltam, MA, USA). Following EU Decision 2020/1729, 11 classes of antimicrobials were included, *i.e.* amikacin and gentamycin (aminoglycosides), ampicillin (penicillin), azithromycin (macrolide), cefotaxime and ceftazidime (cephalosporines), chloramphenicol (phenicol), ciprofloxacin (fluoroquinolone), meropenem (carbapenem), nalidixic acid (quinolone), sulfamethoxazole and trimethoprim (folate pathway antagonists), tetracycline (tetracycline), and tigecycline (glycylcycline).

### Statistical analyses

**Fig. 1:**
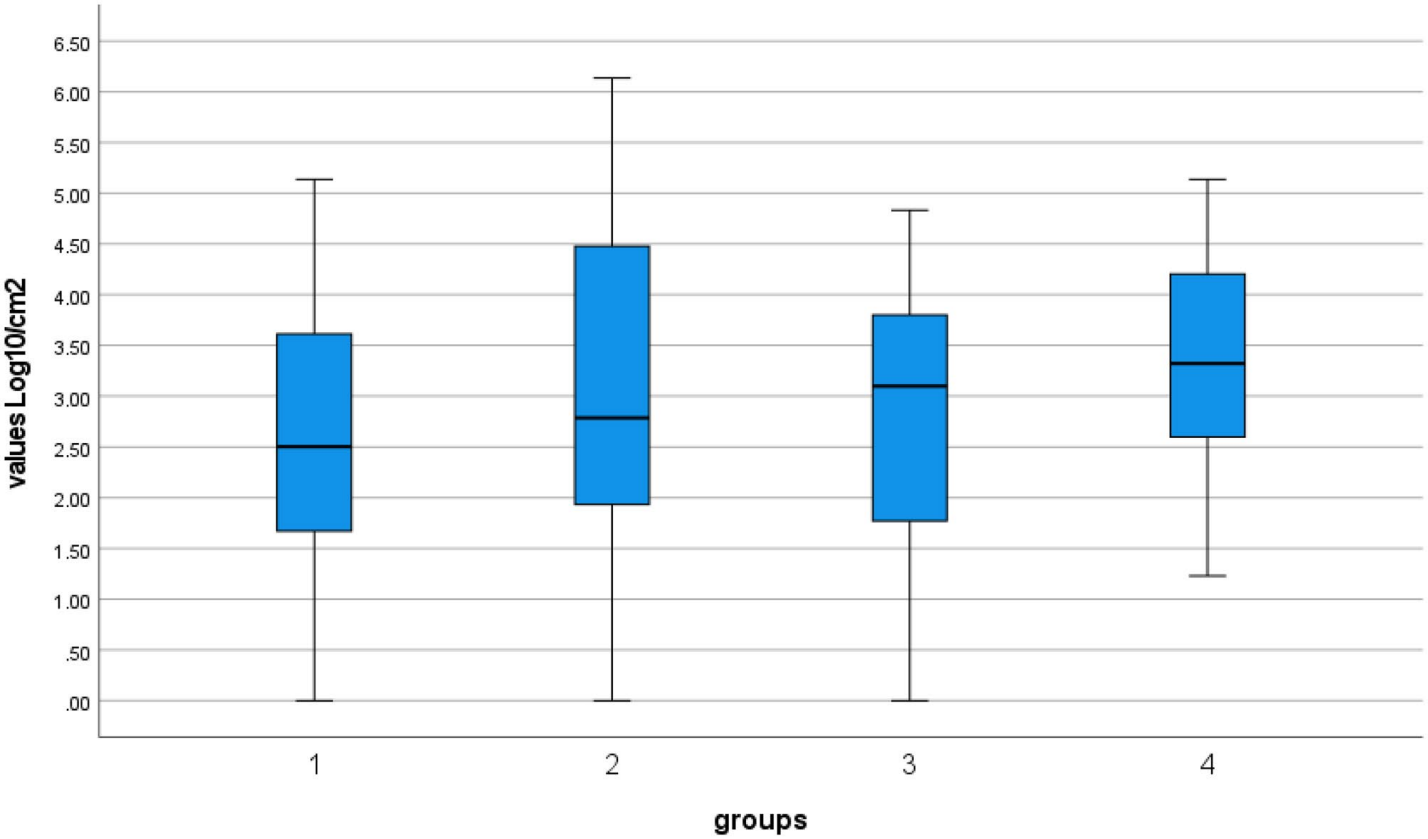
Box-and-whisker diagram of the Enterobacteriaceae count expressed as log_10_ CFU/cm^2^ in the different sampling steps: 1, sampling A1-GCP; 2, sampling A2-GHE; 3, sampling B1-GCP; 4, sampling B2-SH

Enterobacteriaceae were calculated as CFU/cm^2^ and reported converted to log_10_ CFU/cm^2^. Data were analysed using the statistical software IBM SPSS Statistics 27 (IBM, Armonk, NY, USA) and reported in Table 1 and Fig. 1. Statistical differences were analysed with the Wilcoxon signed-rank test. In addition, statistical differences among Enterobacteriaceae count in carcasses shot in the abdomen *versus* other sites (head, neck, heart, thorax) were analysed with Mann–Whitney *U* test. Results were considered statistically significant when *p* < 0.05.

**Table 1:** Median, minimum, maximum and percentiles of Enterobacteriaceae count expressed as Log_10_ CFU/cm^2^ in the different sampling plans

	A1—GCP	A2—GHE	B1—GCP	B2—SH
No	49	49	15	15
Median	2.51	2.79	3.1	3.32
Minimum	0	1.150	0	1.23
Maximum	5.14	6.14	4.83	5.14
25 percentile	1.62	1.78	1.73	2.48
50 percentile	2.51	2.79	3.1	3.32
75 percentile	3.62	4.52	3.88	4.36

A1—GCP: sampling A1 (collection centre). A2—GHE: sampling A1 (game-handling establishment). B1—GCP: sampling B1 (collection centre). B2—SH: sampling B2 (slaughterhouse)

## Results

### Enterobacteriaceae count

The results are described according to the different sampling plans (Table 1 and Fig. 1).

**Sampling A1—GCP** The EBC ranged from 0 log_10_ CFU/cm^2^ to 5.14 log_10_ CFU/cm^2^ (median value 2.51 log_10_ CFU/cm^2^).

**Sampling A2—GHE** The EBC ranged from 0 log_10_ CFU/cm^2^ to 6.14 log_10_ CFU/cm^2^ (median value 2.79 log_10_ CFU/cm^2^). The difference was statistically significant (*p* = 0.001).

**Sampling B1—GCP** The EBC ranged from 0 log_10_ CFU/cm^2^ to 4.83 log_10_ CFU/cm^2^ (median value 3.1 log_10_ CFU/cm^2^).

**Sampling B2—SH** The EBC ranged from 1.23 log_10_ CFU/cm^2^ to 5.14 log_10_ CFU/cm^2^ (median value 3.32 log_10_ CFU/cm^2^). The difference was not statistically significant (*p* = 0. 191).

### Salmonella detection

Ten *Salmonella* isolates were comprehensively detected in the study (Table 2).

**Table 2:** Prevalence of *Salmonella* in carcasses and MLNs at the different sampling sites

	Sampling A (No 49)			Sampling B (No 15)		
	1. Collection center		2. Game-handling establishment	1. Collection center		2. Slaughterhouse
	Carcasses	MLNs	Carcasses	Carcasses	MLNs	Carcasses
*Salmonella* serovar (sample ID)	Agama (CC 32)	Agama (CL 32)				
			Enteritidis (CC 46B)			
		Enteritidis (CL 47)				
		Enteritidis (CL 50)				
			Diarizonae O:50 (z) (CC 53B)			
			Enteritidis (CC 56B)			
		Zaiman (CL 57)				
				Typhimurium (CC 66)		
		Diarizonae O:50 (z) (CL 82)				
Total	1/49 (2.0%)	5/49 (10.2%)	3/49 (6.1%)	1/15 (6.7%)	0/15 (0.0%)	0/15 (0.0%)

**Sampling A1 – GCP** *Salmonella* was isolated from 1/49 carcasses (apparent prevalence 2.0%; 95% CI 0.4–10.7) and from 5/49 MLNs (apparent prevalence 10.2%; 95% CI 4.4–21.8). The carcass isolate belonged to *S. enterica* subsp. *enterica* serovar Agama. The isolates from MLNs were typed as *S. enterica* subsp. *enterica* serovars Enteritidis (two isolates), Agama, Zaiman and *S. enterica* subsp. *diarizonae* O:50 (z). One MLN-positive wild boar (2.0%) ended up as *Salmonella-*positive carcass (*S.* Agama).

**Sampling A2—GHE** *Salmonella* was isolated from 3/49 carcasses (apparent prevalence 6.1%; 95% CI 2.1–16.5). Two isolates belonged to *S. enterica* subsp. *enterica* serovar Enteritidis, and the other one to *S. enterica* subsp. *diarizonae* O:50 (z). No correspondence with the positive results of carcasses and MLNs of Sampling A1 was found.

**Sampling B1—GCP** *Salmonella* was isolated from 1/15 carcasses (apparent prevalence 6.7%; 95% CI 1.2–29.8). The isolate belonged to *S.* *enterica* subsp. *enterica* serovar Typhimurium. MLNs were negative for *Salmonella* spp.

**Sampling B2—SH** *Salmonella* was never detected from the tested carcasses.

### Yersinia enterocolitica detection

*Y. enterocolitica* was never isolated from carcasses or MLNs. Only in sampling A1, 3/49 carcasses were contaminated by bacteria belonging to the genus *Yersinia*. One strain each of *Y. frederiksenii*, *Y. bercovieri* and *Y. aldovae* was detected from carcasses (apparent prevalence for each species 2.0%; 95% CI 0.4–10.7).

### Anatomical shooting location

**Sampling A** Seven of 49 carcasses (14.3%) were shot in the abdomen (group II). The remaining 42 carcasses (85.7%) were shot in the head (20.4%), hearth (4.1%), neck (12.2%), and thorax (49.0%) (group I). The median EBC value was 1.78 log_10_ CFU/cm^2^ for group II and 2.81 log_10_ CFU/cm^2^ for group I (Table 3). The difference between the two groups did not show any statistical difference (*p* = 0.76). *Salmonella* was isolated from one carcass shot in the head (group I) immediately after killing. At the GHE, *Salmonella* was detected in one different carcass shot in the head (group I) and two carcasses shot in the abdomen (group II).

**Table 3:** Median, minimum, maximum of Enterobacteriaceae count expressed as log_10_ CFU/cm^2^ in the expertly shooting (group I) and non-expertly shooting (group II) wild boars in the two sampling plans

	A1—group I	A1—group II	B1—group I	B1—group II
No	42	7	11	4
Median	2.70	1.78	3.39	2.84
Minimum	0	1.15	0	1.81
Maximum	5.14	5.14	4.53	4.83

**Sampling B** Four of 15 carcasses (26.7%) were shot in the abdomen (group II). The remaining 11 carcasses (73.3%) were shot in the head (26.7%), hearth (20.0%), neck (6.7%), thorax (13.3%) and spine (6.7%). The median EBC value was 2.84 log_10_ CFU/cm^2^ for group II and 3.39 log_10_ CFU/cm^2^ for group I (Table 3), without statistical difference (*p* = 0.69). The *Salmonella*-positive carcass belonged to group I (shot in the heart).

### Antimicrobial resistance

One *S. enterica* subsp. *diarizonae* O:50 (z) isolate showed resistance to sulfamethoxazole (MIC  > 512 μg/ml) and sensitivity to the other antimicrobials. The remaining isolates were sensitive to the 14 antimicrobials tested.

## Discussion

In the EU, hunting and processing of wild game are addressed by Regulation No. 853/2004, which recommends evisceration and transportation of large game carcasses to a GHE as soon as possible after killing. Unfortunately, in the territory of the study, the absence of a GHE forced a prolonged chilling (2 °C ± 1 °C) of the skin-on eviscerated carcasses before transportation to the nearest GHE located 150 km away. In these conditions, meat contamination from skin and hide can occur, leading to an increase of the bacterial load of carcasses.

The absence of microbiological criteria for game meat at EU or national level prevents any restrictive action by the CA and does not support FBOs corrective actions in improving poor hygiene conditions. Wild boar slaughtering and handling commonly differ from farmed pig practises, since wild boar carcasses are skinned, while pig carcasses are scalded, dehaired and singed, with the skin remaining in the final carcass. For these reasons, process hygiene criteria values set in the EU for livestock are difficult to extrapolate to wild boar carcasses.

### Enterobacteriaceae count

In our study, we intentionally selected the area at the highest risk of contamination (the abdominal region). This is true not only for non-expertly shooting carcasses but also for each carcass eviscerated out of a proper designed establishment. Gut ruptures, blood, bile and urine shedding, improper hygiene of knives, belly openers and other instruments, contact between carcasses, and use of non-potable water are factors which commonly contribute to microbial contamination of the inner side of the carcasses. EBC values showed a great variation, ranging from 0 log_10_ CFU/cm^2^ to very high microbial loads (i.e. 6.14 log_10_ and 5.14 log_10_ at GHE (A2) and slaughterhouse (B2), respectively). To describe our results, we preferred using the median values instead of the mean values, because of the great dispersion of data. In addition, we were not interested in the comparison with the mean values proposed by the EU Regulations for farmed animals, since their pre-harvest and harvest operations are not comparable. As reported in Table 1, the median EBC in the interior carcass meat surface ranged from 2.51 log_10_ CFU/cm^2^ (GCP) to 2.79 log_10_ CFU/cm^2^ (GHE) in sampling A and from 3.1 log_10_ CFU/cm^2^ (GCP) to 3.32 log_10_ CFU/cm^2^ (SH) in sampling B. Enterobacteriaceae rarely grow at refrigeration temperature, but this ability on meat was demonstrated for strains of *Hafnia alvei* (2.6 °C), *Serratia liquefaciens* (1.7 °C), *Enterobacter agglomerans* (1.3 °C) and *Serratia fonticola* (2.0 °C) (Ridell and Korkeala 1997). Likely explanations of the increase in the microbial contamination of carcasses in both sampling trials can be found in the presence of bacterial species capable of growth at refrigeration temperature, in the variation of temperature due to the periodical introduction of freshly hunted carcasses into the chilling room, as well as in the transport conditions. In sampling A, the median EBC increase between carcasses tested at the GCP and the GHE was statistically significant (*p* = 0.001), as opposed to sampling B (*p* = 0.191). Since the average time interval between animal evisceration and carcass processing varied from 4.3 days (sampling A) to 2.3 days (sampling B), we recommend shortening it as soon as possible, as stated in the EU legislation.

However, beside the statistically significant microbial increase related to the time interval between shooting and transportation to the GHE, the initial microbial load of wild boar carcasses (A1, B1) had a great impact on final EBC values (A2, B2). For this reason, the respect of good hygienic practices during evisceration by hunters is crucial in preserving the microbial quality of carcass meat. Some depreciable practises should be completely avoided, such as evisceration of carcasses in lying position on the ground in the field or on the floor in collection centres, not removal of blood, soil, hair, and organic matter from the cavities of the carcasses, as well as use of contaminated equipment and non-potable water.

The comparison with other studies is not easy, especially because of the variations in sampling strategies. Furthermore, most studies were performed on freshly shot carcasses (Atanassova et al. 2008; Avagnina et al. 2012; Mirceta et al. 2017), thus focusing the attention on the primary production step, which is only the first link in the complex game meat chain.

In Germany, the destructive method was used to collect four tissue samples of ≤ 5 mm thickness from different sites (total surface area 20 cm^2^) of 127 wild boar carcasses. Sampling took place at a central collection point, approximately 2 h after hunting. The EBC varied between 1.7 and 3.5 log_10_ CFU/cm^2^, and the mean value was 2.1 log_10_ CFU/cm^2^ (Atanassova et al. 2008). In Serbia, 210 wild boar carcasses were swabbed in four sites of 100 cm^2^ each (i.e. interior surface of the rump and flank, thorax and brisket) soon after evisceration. The authors reported a mean EBC of 3.8 log_10_ CFU/cm^2^ (Mirceta et al. 2017). An Italian study investigated the microbiological conditions of 72 wild boar carcasses within 1–6 h after shooting; after their arrival at the collection site, each carcass was swabbed by hunters themselves in areas of 25 cm^2^ within the region of the medial hindlimb (*mm. semitendinosus/semimembranosus*). The mean EBC value was 3.0 log_10_ CFU/cm^2^ (Avagnina et al. 2012).

A different study was performed in Italy, during which the microbial load of wild boar carcasses was assessed after dressing and processing at the GHE. The microbial contamination of 62 wild boar carcasses transported to an establishment 1 to 3 h after hunting was evaluated. After slaughtering operations (skinning, evisceration, and half-carcass sectioning), non-destructive samplings were performed by swabbing four areas of 100 cm^2^ on the skinned exterior part of the carcasses, following the sampling locations proposed by Decision No. 471/2001 for cattle (rump, flank, brisket and neck). The mean EBC value was as low as 1.32 log_10_ CFU/cm^2^, and for most sampling sessions, the mean log values were below the highest threshold set by Regulation No. 2073/2005 for cattle (Stella et al. 2018). Such testing was representative of the Enterobacteriaceae load of the carcass meat before any other processing operations (sectioning, trimming, packaging, chilling) and was performed at the GHE, thus being similar to carcass sampling of the other food-producing animals covered by Regulation No. 2073/2005. On our opinion, sampling of wild game carcass should be always performed at the GHE rather than after shooting, because the microbiological features of freshly shot carcasses could be further influenced by additional factors (storage time, chilling temperature, persistence of blood clots, gut content, organic detritus, etc., as well as hygienic conditions of trucks). Anyway, we strongly suggest including a sampling area covering the inner part of the carcass (flank) to reflect the microbiological status of carcasses eviscerated by hunters and sometimes affected by bowel rupture, organ laceration, traces of hair and soil, as well as blood, bile, and urine shedding.

### Bacterial pathogens

Similar to EBC, *Salmonella* contamination of the interior site of the carcasses varied after a prolonged storage at the GCP. In sampling A, the carcass which was found *Salmonella*-positive at the  GCP was negative at the GHE. Nevertheless, the prevalence of positive carcasses at the GHE was three-fold higher (3/49; 6.1%) if compared with the freshly shot carcasses (1/49; 2.0%) (Table 2). *Salmonella* contamination can be enhanced by the storage of skin-on carcasses, as demonstrated by the detection of the pathogen from the skin of wild boars (Mirceta et al. 2017). Interestingly, EFSA suggests testing pig carcasses for *Salmonella* prior to chilling rather than after chilling because bacterial active attachment to the carcass surface and bacterial stress due to the low temperature are two factors that reduce the pathogen recovering (EFSA 2011). We can extrapolate this concept to wild game meat carcasses and suppose that additional contamination of the carcasses transported to the GHE should have occurred during their prolonged storage. On the contrary, in sampling B, *Salmonella* was detected in freshly shot carcasses only (1/15; 6.7%) and not after chilling, thus confirming EFSA’s opinion.

Detection of *Salmonella* from wild boar carcasses is not common, especially when the animals do not share pastures with livestock (Avagnina et al. 2012). Other surveys did not recover *Salmonella* from wild boar carcasses (Atanassova et al. 2008; Avagnina et al. 2012) or found low prevalence (1.9%) (Mirceta et al. 2017). In our study, wild boar carcasses were contaminated by *S.* Agama, *S.* Enteritidis, *S.* Typhimurium and *S.* *enterica* subsp. *diarizonae*O:50 (z), while from MLNs *S.* Agama, *S.* Enteritidis, *S.* Zaiman and *S.* *enterica* subsp. *diarizonae* O:50 (z) were found.

*S.* Enteritidis and *S.* Typhimurium are the most frequent serovars in human cases of salmonellosis in Europe, representing 50.3% and 11.9% of the reported serovars in 2019, respectively (EFSA and ECDC 2021). These serovars have been detected in wild boars in different European countries, commonly with low prevalence (Bonardi et al. 2019; Chiari et al. 2013; Gil Molino et al. 2019; Sannö et al. 2014) except in Portugal, where 22% of faecal samples were positive for *S.* Typhimurium (Vieira-Pinto et al. 2011). *S. enterica* subsp. *diarizonae* was commonly found in wild boars in Italy, although with variations in the antigenic formula (Chiari et al. 2013; Zottola et al. 2013). On the contrary, to the best of our knowledge, this is the first report of *S. enterica* subsp. *enterica* serovars Agama and Zaiman from wild boars in Europe. *S.* Agama was detected in badgers and cattle in Great Britain (Davies et al. 2004; Wilson et al. 2003) as well as from rainbow lizards (*Agama agama*) and humans in Africa (Bélard et al. 2007). *S.* Zaiman is a rare serovar, first isolated from human patients and Zaiman river in Argentina (Vergara et al. 1989). Recently, it has been isolated from red foxes (*Vulpes vulpes*) in Italy, thus demonstrating its occurrence in wild animals (Rubini et al. 2016). Since information on rare serovars are difficult to collect, our data might be useful in the epidemiological studies on human cases of salmonellosis caused by uncommon *Salmonella* strains.

*Y. enterocolitica* was never isolated from carcasses or MLNs. This finding is not surprising, as wild boars are not good reservoirs of human pathogenic *Y. enterocolitica* in the study area (Bonardi et al. 2020), as opposed to central Italy (Cilia et al. 2021) or other European countries (Fredriksson-Ahomaa et al. 2011; Sannö et al. 2014; Sannö et al. 2018; Syczylo et al. 2018). The other *Yersinia* species detected on carcasses (*Y. frederiksenii*, *Y. bercovieri* and *Y. aldovae*) are not considered agents of human yersiniosis (EFSA and ECDC 2021).

### Anatomical shooting location

In our study, the proportion of non-expertly shot animals (i.e. in the abdominal region) was rather low (11/64; 17.2%) if compared to other studies, where 21%, 35% and 43% of the wild boars were shot in the abdomen (Atanassova et al. 2008; Avagina et al. 2012; Mirceta et al. 2017). Commonly, the shot location in the abdomen is followed by an increase of ACC and EBC of carcasses, if compared to expertly shot animals (Atanassova et al. 2008; Avagina et al. 2012; Mirceta et al. 2017). Avagina et al. (2012) reported that high microbial loads in 30% of the animals were associated with visible contamination of the carcass with soil or gut content. Mirceta et al. (2017) observed that the EBC could be 0.8 log_10_ CFU/cm^2^ higher in non-expertly shot animals, but believed that many factors could overshadow the impact of abdominal shot on the microbial contamination of carcasses, as poor hygiene during evisceration, evisceration on the ground, and skin washing. On the contrary, we observed that the carcasses shot in the abdomen showed lower median EBC values compared to expertly shot carcasses. Even if the difference in median EBC values was not statistically significant, it is rather surprising that correct shot placements were associated to higher microbial counts. In parallel, *Salmonella* could not be detected immediately after evisceration in group II carcasses, as opposed to group I carcasses. Prevalence of *Salmonella* in group I (53 carcasses) was 3.8% (2/53), thus confirming that the abdominal shooting location is not the only factor influencing hygiene and safety of hunted animals.

A possible explanation for the lower microbiological contamination of group II in comparison to group I could be found in the washing step, which was particularly accurate when the wild boars were shot in the abdominal region. Indeed, the practice of interior carcass washing was routinely applied after evisceration and bleeding. This is commonly considered an unhygienic practice, able to create contaminated aerosol which can lead to a significant increase in ACC and EBC on carcass meat (Mirceta et al. 2017). Anyway, washing was used to remove blood clots and—when present—faecal material or soil from the carcass cavities, thus preventing microbial growth during the prolonged chilling storage before processing at the GHE. The skin was not washed, thus avoiding any contamination from the external to the internal surface of the carcasses. For these reasons, we did not reject interior carcass washing procedure itself if other hygienic measures are assured and trained staff is employed. Faecal matter, blood, soil, hair should be rapidly removed from the inner cavities of wild game carcasses to avoid rapid spoilage and putrefaction of meat. The washing step should be performed in a collection structure only and not in the field, where hunters often use water from rivers or streams to remove visible contamination from freshly shot carcasses. On the contrary, hunters should be instructed to prefer a well-performed washing step to preserve the quality of game meat. In fact, since hunters are designed by the EU legislation as primary producers, they should be aware of the duties and responsibilities they have in the game meat chain.

### Salmonella antimicrobial resistance

As well known, sensitivity to antimicrobials in *Salmonella* might support any therapies patients should need. In our isolates, AMR was restricted to one sulfamethoxazole-resistant isolate of *S. enterica* susps. *diarizonae* O:50 (z). The remaining isolates (88.9%) were sensitive to the 14 antimicrobials tested, independently from their serovars, in accordance with previous results obtained in the same Italian region (Bonardi et al. 2019; Rossi et al. 2007). Since contamination by AMR bacteria in wild animals is commonly attributed to environmental circulation of microorganisms originating from farmed animals or anthropic sources, our findings suggest a close segregation between wild boar population and farmed food-producing animals, as already observed comparing AMR of *Salmonella* strains isolated from farmed pigs and wild boars in the study area (Bonardi et al. 2019).

## Conclusions

Since wild boar hunting and slaughtering are completely different from livestock farming and slaughtering, extrapolation of EU process hygiene criteria to wild game is challenging. In our opinion, process hygiene criteria at the GHE for wild game are needed and should be included in the EU legislation, to guarantee hygiene and safety of the entire game meat chain.

According to our results, EBC is a crucial microbiological parameter for chilled carcasses processed at the GHE, reflecting the hygienic status of wild boar meat entering the food chain. In our study, EBC increased significantly when carcass processing at the GHE was delayed (average time from hunting 4.3 days). In parallel, *Salmonella* prevalence increased, probably due to contamination deriving from the skin. Even if the results are referred to a limited number of carcasses, we suggest avoiding long storage periods prior to wild game de-hiding and processing at the GHE. Hunting territories should be equipped with quickly reachable GHEs to ensure high hygienic and safety standards of wild game meat, which nowadays represents a sustainable, organic, antibiotic-free and high-quality food resource for the consumers.
